# Influencing Elimination Location in the Domestic Cat: A Semiochemical Approach

**DOI:** 10.3390/ani12070896

**Published:** 2022-03-31

**Authors:** Naïma Kasbaoui, Cécile Bienboire-Frosini, Philippe Monneret, Julien Leclercq, Estelle Descout, Alessandro Cozzi, Patrick Pageat

**Affiliations:** 1 Animal Behaviour and Welfare Department, Research Institute in Semiochemistry and Applied Ethology (IRSEA), Quartier Salignan, 84400 Apt, France; 2 Molecular Biology and Chemical Communication Department, Research Institute in Semiochemistry and Applied Ethology (IRSEA), Quartier Salignan, 84400 Apt, France; c.frosini@group-irsea.com; 3 Animal Experimentation Service, Research Institute in Semiochemistry and Applied Ethology (IRSEA), Quartier Salignan, 84400 Apt, France; p.monneret@group-irsea.com (P.M.); j.leclercq@group-irsea.com (J.L.); 4 Data Management and Statistics Service, Research Institute in Semiochemistry and Applied Ethology (IRSEA), Quartier Salignan, 84400 Apt, France; e.descout@group-irsea.com; 5 Research and Education Board, Research Institute in Semiochemistry and Applied Ethology (IRSEA), Quartier Salignan, 84400 Apt, France; p.pageat@group-irsea.com

**Keywords:** animal communication, animal behaviour, elimination behaviour, elimination location, human–animal interactions

## Abstract

**Simple Summary:**

In the domestic cat, elimination at an inappropriate location is considered by cat owners and non-cat owners as an undesirable behaviour. The aim of the study was to assess the effects of a composition derived from cat anal glands on the elimination behaviour of domestic cats in a cattery setting. The study was conducted in four catteries housing 33 cats, using 37 litter trays. The data collection lasted two weeks, each litter tray receiving one treatment on the first week and the other treatment on the second week. The parameters studied included daily elimination (urine plus stools) weight, urine weight, stool weight, elimination type and urine/stool quantity scoring. Four out of the six parameters studied showed a treatment effect, consistently in favour of cats defecating significantly less in the litter trays sprayed with the treatment versus litter trays sprayed with the control. These results demonstrate that a composition originating from cat anal glands influences cats’ defecation location.

**Abstract:**

In the domestic cat, elimination at an inappropriate location is considered by cat owners and non-cat owners as an undesirable behaviour. The aim of the study was to assess the effect of a semiochemical formulation, reconstituted volatile fraction of cat anal gland secretions on the elimination behaviour of domestic cats. The study was conducted in four catteries, which housed 33 cats, using 37 litter trays and followed a randomised crossover design using the litter tray as the experimental unit. The parameters studied included daily elimination (urine plus stools) weight, urine weight, stool weight, elimination type and urine/stool quantity scoring. The parameters were analysed using GLMM with SAS 9.4 software. Four out of the six parameters studied showed a treatment effect, consistently in favour of cats defecating significantly less in the litter trays sprayed with the treatment versus litter trays sprayed with the control (elimination weight *p* = 0.0199; elimination type *p* = 0.0251; stool weight *p* = 0.0005 and stool quantity *p* = 0.003). These results demonstrate that an intraspecific semiochemical message originating from cat anal glands influences cats’ defecation location.

## 1. Introduction

Animals use various methods to gather information about their physical and social environment to secure the resources necessary for survival. They explore their physical environment and gather information about other animals, especially conspecifics, using means of communication. Communication can be defined as the transmission of information from one animal to another [1] using different sensory modalities, such as visual, auditory, and chemical signals [2,3]. Chemical signals are emitted towards a receiver, either in the same species or another species [4]. Chemical communication allows the animal to gain information about its environment and other animals, even when the animals are not present [5]. The domestic cat as a species has a flexible social life, where they may live in a colony but also as a single individual, with conspecific encounters not being very frequent [5]. Chemical communication is thus an essential way of gathering information about conspecifics in the environment. Research has shown that urine, faeces, and glandular secretions can be vectors of chemical communication [6,7,8,9], and chemical compounds have been identified in urine [10,11], faeces [12,13] and anal glands. In particular, secretions from anal glands are used for scent communication in a number of species (honey badger (*Mellivora capensis*)) [14], red foxes (*Vulpes vulpes*) [15,16,17,18], spotted hyena (*Crocuta crocuta*) [19], either by being directly deposited on a number of substrates (spotted hyena [20]) or by being excreted in urine [10] and/or on faeces [7]. The exploration of the role of these compounds is an expanding research field, as they could be used for individual recognition [12], reproductive advertisement [10], or territorial marking. Linking compounds to a specific role (for example, in cats [8]) to better understand the interaction between chemical compounds and bacteria present in the glands [21] or the interaction between compounds and their binding proteins [22] are also avenues of research.

Cat elimination behaviour is a complex process [23] that fulfils the role of excreting waste from the body and is also used as a means of communication with conspecifics, providing information about species, sex, reproductive status of conspecifics and age for male cats [12,13]. Additionally, similar to other forms of chemical communication, such as scratching [24] and rubbing, it can be used to scent mark the cat’s territory, thus influencing the behaviour of other cats. Urinary extracts have been shown to influence the choice of location to eliminate in outdoor cats [25], and while some of the roles of chemical compounds isolated from cat anal glands have been explored [8], there is a lack of knowledge about the relationship between chemical compounds and the influence they can have on cat choice of location to eliminate. The aim of our study was to assess the effect of a semiochemical formulation, that is, a reconstituted volatile fraction derived from cat anal glands, on the elimination behaviour of domestic cats in a cattery model.

## 2. Materials and Methods

### 2.1. Animals and Housing Conditions

**Figure 1:**
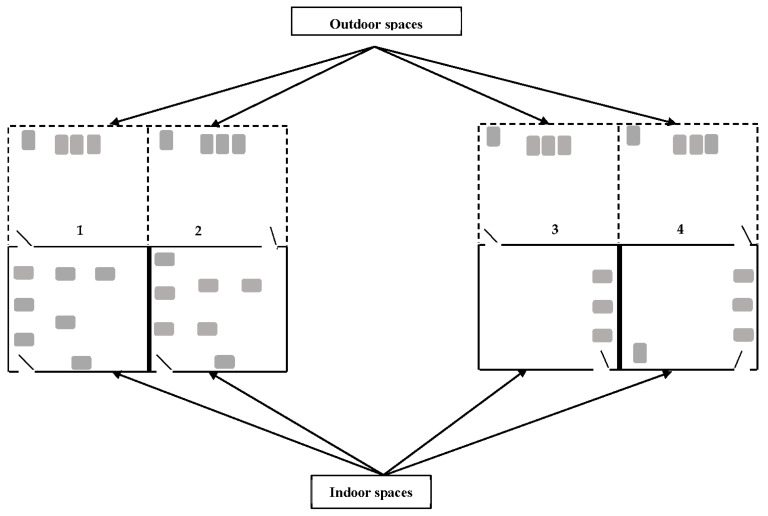
General layout of the litter trays in the catteries for the study. The litter trays are depicted as grey rectangles. Cattery 1: entire female cats, cattery 2: entire male cats, cattery 3: senior cats, cattery 4: overweight cats. Litter trays are placed as far as possible from each other and following space constraints.

The study was conducted on 33 cats (10 entire males, 10 entire females, 5 neutered males and 8 neutered females) in their usual housing catteries. The cats were familiar with each other and had been living together for at least 20 months at the time of the study. Cats were examined by a veterinarian at the beginning of the study and were found to be in good health, free from chronic diseases or urinary tract diseases. The cats were distributed based on sex, age and weight in four catteries (cattery 1: entire female cats, cattery 2: entire male cats, cattery 3: senior cats, cattery 4: overweight cats, see Table 1); the catteries consisted of an indoor (12.72 square metres) and an outdoor (12.23 square metres) space, both spaces linked by cat flaps. The catteries were equipped, on the same model, with platforms, beddings, hiding places, food bowls and toys in enough numbers so that the cats could have their own resources and in fixed positions to avoid any confounding effects. Water was provided ad libitum, and dry food was provided to match the specific needs of each cattery (Royal Canin Kibble VCN Adult Cat, VCN Senior Cat Stage 2 and Cat Satiety) in a quantity sufficient for maintaining an adequate weight. The cats had free roam of both spaces at all times. Following the N+1 recommendation (N+1 litter tray per cattery, N being the number of cats per cattery [26]), 37 litter trays, identical in shape and size (Savic Aseo litter tray, L56 cm × l39 cm × H27.5 cm) were distributed throughout the catteries to maximise individual litter tray use. The distribution within the catteries was made so that the litter trays were away enough from each other so that each litter tray could be chosen at any moment even if another cat was using the closest litter tray and following space constraints (Figure 1). For example, in the outdoor spaces, only four litter trays could be fitted taking into account the catteries’ layout. Each litter tray was filled with 1.5 kg of non-agglomerant litter substrate of the brand “Prop’chat NF”.

### 2.2. Experimental Procedure

#### 2.2.1. Experimental Design and Treatments Applied

The chosen experimental design was a crossover, with the litter tray as the experimental unit, to minimise the confounding factors in the comparison of treatments applied [27]. The litter substrate inside the litter tray was sprayed with two types of treatments, one treatment at a time. One treatment was the reconstituted volatile fraction (named CEMS for Cat Elimination Modulation Semiochemical) derived from an entire male cat’s anal gland secretion, diluted at 2% in a mix of ethanol and water (60% ethanol). After the cat anal gland secretion of an entire male cat was extracted and its compounds identified, the treatment CEMS was synthetised and diluted in the solvent. The other treatment was the control (i.e., a mixture of ethanol and water, with 60% ethanol).

The procedure was blinded, so the operators only knew the treatments as A and B. During the experiment, treatments were randomly assigned to the litter trays, taking into account that in each cattery, approximately half the litter trays available contained A and the other half contained B, to ensure that the cats had the choice between the two treatments. For one week, on four days per week, each litter tray received one of the two treatments (for example, A), randomly assigned, and then, during the second week, the same litter tray received the other treatment (B), according to the crossover design. Litter trays were filled with 1.5 kg of litter substrate and weighed at the start of the experiment (Expondo digital weighing scale, model SBS-PT-40/1, with a precision of 1 g). Then, twice a day (9 a.m. and 3 p.m.), each litter tray was removed from the cattery and weighed. The type of elimination present was recorded (urine only, stools only, or urine plus stools), and scoring of the urine spots and stool production was performed (i.e., counting the urine spots and the stool piles in the litter tray and attributing a score, 0 for “no urine spots or stool piles”, 1 for “one urine spot or stool pile”, 2 for “two to three urine spots or stool piles” and 3 for “more than three urine spots or stool piles”). The stools were then removed if present, and the litter tray was weighed again to measure the weight of the urine only. After all the data were recorded, the litter substrate was discarded in a special bin. For cleaning, the litter tray was sprayed on the inside and the outside with a detergent disinfectant product that destroys liposoluble compounds (DNA02 LeVrai Professionel, https://www.bernard.fr/, accessed on 29 October 2021) and thoroughly wiped until completely clean. Then, it was sprayed a second time with water to rinse the detergent to avoid any contact between the detergent and the cats. After the litter tray was dried, 1.5 kg of fresh litter substrate was weighed and placed in the litter tray. Finally, according to the randomisation list, the designated treatment was sprayed (five sprays) on the litter substrate and mixed with it. The litter tray was weighed to record the weight of the unused litter tray with the treatment applied and was put back in place. Data collection was performed throughout the whole experiment by the same operator. The parameters studied included total elimination weight (urine plus stools, which was calculated by subtracting the weight of the unused litter tray with the treatment from the weight of the used litter tray), urine weight (which was calculated by subtracting the weight of the unused litter tray from the weight of the used litter tray without stools), stool weight, type of elimination, and score of urine spots and stools. It was not possible to collect data via video regarding cat elimination because the litter trays were distributed throughout the catteries, mostly below shelves, which makes it technically impossible to see if the cat is urinating or defecating.

#### 2.2.2. Effect of Climatic Conditions

To check the influence of climatic conditions on the litter substrate and on the litter trays’ weight, the weight difference was also recorded when the litter tray was unused between two data points. Temperature and humidity were also recorded in each cattery, in the indoor space and the outdoor space, twice a day. The study was carried out in July in hot and dry weather.

### 2.3. Statistical Analysis

The data were analysed using SAS 9.4 software (Copyright (c) 2002–2012 by SAS Institute Inc., Cary, NC, USA), and the significance threshold was fixed at 0.05.

For the continuous variables (weights), the normality of residuals from the raw data was first verified with the UNIVARIATE procedure. Normality was not verified, so a box-cox transformation was applied to total elimination and stool weight and a log transformation was used for the parameter urine weight to obtain the normality of residuals. Then, the effects of treatment (A and B), week (1 and 2), sequence (A/B and B/A), litter tray location (inside and outside), day (1, 2, 3 and 4) and cattery (1: male cats, 2: female cats, 3: senior cats and 4: overweight cats) were assessed using a General Linear Mixed Model, with the MIXED procedure (litter tray was considered a random effect). The best option in the model simplification was selected according to the fit statistics (AICC and BIC). Multiple comparisons for significant differences were analysed with the TUKEY test using the LSMEANS statement in the MIXED procedure with the option ADJUST = TUKEY. For the three polytomous variables (type of elimination, scores of urine spots and stools), a Generalized Estimating Equation Model was used with the GENMOD procedure, specifying the MULTINOMIAL DISTRIBUTION and the CUMLOGIT link function to assess the effects of product, week, sequence, litter tray location, day and cattery. The best option in the model simplification was selected according to the fit statistics (QIC and QICu). After statistical analysis was carried out, the blinding was lifted: A was the control treatment and B the CEMS treatment.

## 3. Results

The sequence effect (effect of the treatment depending on the order of administration) was tested to determine whether the crossover design was valid. There was no effect of sequence on any of the parameters tested, which means that the order of administration and the effect of the treatments were independent. Moreover, the effect of treatment and litter tray location interaction was tested in the model. There was no significant effect on any of the parameters tested, which allowed the removal of the interaction from each model.

### 3.1. Weight of Elimination

For the total elimination weight, we observed a significant effect of **treatment** (GLMM; Num DF = 1; Den DF = 73.2; F = 5.66; ***p* = 0.0199**), **week** (GLMM; Num DF = 1; Den DF = 73.2; F = 6.98; ***p =* 0.0101)** and **litter tray location** (GLMM; Num DF = 1; Den DF = 32; F = 15.94; ***p =* 0.0004**) (Table 2).

For urine weight only, we observed a significant effect of **week** (GLMM; Num DF = 1; Den DF = 181; F = 5.71; ***p* = 0.0179**), **litter tray location** (GLMM; Num DF = 1; Den DF = 31; F = 14.38; ***p* = 0.0006**) and **cattery** (GLMM; Num DF = 3; Den DF = 31; F = 2.92; ***p* = 0.0497**) with the overweight cats (cattery 4) urinating significantly more than the entire male cats (cattery 2; DF = 31; t = 2.73; ***p* = 0.0497**) (Table 2).

For the stool weight, we observed a significant effect of **treatment** (GLMM; Num DF = 1; Den DF = 35; F = 14.77; ***p* = 0.0005**) and **litter tray location** (GLMM; Num DF = 1; Den DF = 32; F = 12.73; ***p* = 0.0012**).

### 3.2. Type of Elimination and Elimination Scoring

For the type of elimination, we observed a significant effect of **treatment** (GEE; χ2 = 5.02; DF = 1; ***p* = 0.0251**) and **litter tray location** (GEE; χ2 = 7.40; DF = 1; ***p* = 0.0065**) (see Table 3).

For the scoring of stools, we observed a significant effect of **treatment** (GEE; χ2 = 8.80; DF= 1; ***p* = 0.0030**) and **litter tray location** (GEE; χ2 = 9.59; DF = 1; ***p* = 0.0020**) (see Table 3).

Finally, for the scoring of urine spots, we did not observe any significant effect. Considering the results for all the parameters, the cats defecated significantly less in the litter tray where the CEMS treatment was applied than in the litter tray where the control treatment was applied.

## 4. Discussion

The aim of the study was to assess the effect of a synthetic semiochemical formulation, reconstituted volatile fraction of cat anal gland secretionson the elimination behaviour of domestic cats, specifically their choice of location to eliminate. The study was carried out in a cattery setting, mimicking a multi-cat household.

We chose a crossover design, with the litter tray as an experimental unit, to reduce the potential cattery effect [27]. The results showed that cats defecated significantly less in the litter tray where the CEMS treatment was applied than in the litter tray where the control treatment was applied.

Scent marking by carnivores can be performed using urine, faeces and sebaceous glandular secretions [6,7]. Anal gland secretions are excreted on faeces [8]. Cats usually bury their faeces, especially in their core home range, but it has been reported that sometimes, in the peripheral area of their home range, the faeces are left exposed [7]. This behaviour seems more frequently exhibited by entire male cats. In addition, it has been recently shown that faeces can be a chemical basis for species, sex and individual recognition in domestic cats [12] and aging in male cats [8,13], and that the chemical profiles of anal gland compounds are highly conserved within individuals [8]. Our hypothesis is that anal gland secretions that are excreted on the faeces could serve as a territorial scent marking and therefore may be deterring other cats from depositing faeces in the same area. This could explain the observations made in this study. Our results contrast with a recent study on the influence of the “previous use of the litter tray” on cat elimination preferences, which did not find an effect of faeces odour on cat preferences [28]. However, there are differences in the substances’ sample preparation between the two studies. In Ellis and colleagues’ work, the faeces sample consisted of homogenised water plus the faeces of a familiar cat. In our study, the molecules were dissolved in a mix of ethanol and water because some molecules are not soluble in water. We can therefore make the hypothesis that the molecules that were presented in the two studies were different, or at least evaporated in a different way, which could explain the differences between the two studies.

A treatment effect was not observed in the parameters directly related to urine production (urine weight and scoring of urine spots), which is different from the results of a recent study that used feline urine extracts to assess the possibility of managing free roaming cats’ toileting behaviour [25]. There were two main differences between the studies. Miyazaki and colleagues used feline urinary extracts in an outdoor setting where cats would most likely individually pass and explore the odour that was presented, while our study used a reconstituted volatile fraction derived from cat anal glands in a cattery setting, mimicking a multi-cat household. Social conflict between cats is known to influence the choice of location to eliminate or trigger out-of-litter-tray elimination [29]. It is possible that in our setting, with cats urinating more times per day than defecating, social conflict may have pushed some cats to eliminate in litter trays with the treatment. Additionally, urinary extracts are very likely to be different from anal gland compounds [8,10,13,30]; hence, the effects of the two formulations tested may be very different.

A significant effect of week was shown on several parameters, such as the total elimination and stool only weight variables, but not on the type of elimination or the stool scoring parameters, which implies that while the cats did not change their elimination behaviour, the weight of the elimination changed. We examined the temperature and humidity in the two weeks, and if the temperature was very similar between the two periods, the humidity varied from day to day, one constant being that recorded humidity in the outdoor spaces was lower than in the indoor spaces, due to the climatic conditions. However, when we studied litter trays that were not used for 24 h and compared their weight difference per 24 h, the increase in weight was nearly the same, differing by only a few grams. The precision of our weighing scale was one gram, and the humidity variations could not explain the period effect. Litter tray location is an important factor in the choice of litter tray to eliminate [29]. There were more unused litter trays in week 2 than in week 1, and the difference in the weight of empty litter trays between the treatment and the control increased in week 2. Therefore, this significant effect of week could be explained by several factors reinforcing each other, such as the treatment randomly sprayed on litter trays that were already used less because of their location.

A cattery effect on urine weight was observed, wherein overweight cats (cattery four) had a higher urine weight than male cats (cattery two). This effect was observed on one parameter only, that is, the one that characterises cat urination. Due to the validated crossover design, this could not be explained by a building effect. The largest weight difference was observed in cattery four, containing cats that tended to be overweight. As urine production can be influenced by cat weight [31], we analysed the average weight of each cattery. There were significant differences: cattery four and cattery two (overweight cats and male cats, respectively) weighed significantly more on average than cattery one (female cats), but this did not explain the difference between male cats and overweight cats with regard to urine weight. However, the “male” cattery (cattery one) housed only entire males, which displayed spraying behaviour at a higher frequency than cats in the other catteries [32]. Therefore, the fact that the urine weight was the lowest in the “male” cattery could be linked to the fact that they may have sprayed outside the litter trays and therefore, some urine data could not be collected in this cattery.

Finally, a significant effect of the litter tray location (inside versus outside) was observed on four out of the five parameters studied (all but the scoring of urine, which showed no effect at all), and in all cases, the inside litter trays were used significantly more. However, it was shown in this study that the inside litter trays’ daily weight increase when they were not used was two times higher than the outside litter trays’ daily weight increase. Therefore, one hypothesis is that the difference in daily weight increase explains the effect observed on the position of the litter tray. However, the three other parameters, including the type of elimination and the stool scoring, which do not depend on humidity, showed the same effect. Another hypothesis is that the number of litter trays inside, for technical reasons and to follow the N+1 recommendation (N being the number of cats), could be a confounding factor, as there were many more litter trays inside in two catteries out of the four. One final factor may be able to explain this effect: weather conditions. Our study was carried out in July, with heat waves and outside temperatures above 30 °C every day, while the inside space had a regulated temperature. The cats’ elimination behaviour could have been influenced by this factor.

## 5. Conclusions

In a cattery model mimicking a multi-cat living environment, we demonstrated that a semiochemical formulation, the reconstituted volatile fraction of an anal gland extract, influenced the choice of location to defecate in domestic cats, deterring the cats from defecating in the location where the formulation was present. These results are of interest for better understanding how cats communicate with their conspecifics. In an applied setting, this formulation could help manage unwanted toileting in free-roaming cats, which could improve the relationship between cats and the human community. Further research is warranted to confirm this effect on individual cats and better understand why the choice of location to urinate was not affected during our study.

## Figures and Tables

**Table 1:** Distribution of cats in catteries, cats’ sexes and ages.

Cattery	Cat Name	Cat Sex	Cat Age
1	Altesse		
	Biscotte		
	Brioche		
	Choco		
	Cannelle	Entire female	2 years 4 months
	Gaia		
	Mia		
	Xarra		
	Xena		
	Venus		
2	Matisse		
	Merlin		
	Misty		
	Next		
	Simba	Entire male	2 years 4 months
	Spicy		
	Willy		
	Woody		
	Ying		
	Yang		
3	Batcat	Neutered male	13 years 1 months
	Ecaille	Neutered female	11 years
	Edelweiss	Neutered female	11 years
	Hermine	Neutered female	7 years 4 months
	Perle	Neutered female	14 years 3 months
	Rose	Neutered female	14 years 3 months
4	Barbouille	Neutered male	13 years 1 months
	Corona	Neutered female	8 years
	Elvira	Neutered female	11 years
	Encre	Neutered male	13 years
	Garfield	Neutered male	8 years
	Guimauve	Neutered female	8 years 2 months
	Guinness	Neutered male	8 years

**Table 2:** Effect of treatment, week, litter tray location and cattery, on the total elimination weight, urine only weight and stool weight (significant differences are highlighted in **bold**).

Variable	Effect		Mean	SE	DF	F	*p*-Value
Total elimination weight (kg)	Treatment	Control	0.093	0.005	1	5.66	**0.0199**
		CEMS	0.084	0.005			
	Week	1	0.092	0.005	1	6.98	**0.0101**
		2	0.085	0.005			
	Litter tray location	Inside	0.108	0.005	1	15.94	**0.0004**
		Outside	0.063	0.005			
	Cattery	Senior	0.089	0.010	3	2.29	0.0975
		Overweight	0.125	0.009			
		Female	0.073	0.005			
		Male	0.078	0.005			
Urine weight (kg)	Treatment	Control	0.069	0.004	1	1.28	0.2590
		CEMS	0.065	0.004			
	Week	1	0.070	0.004	1	5.71	**0.0179**
		2	0.064	0.004			
	Litter tray location	Inside	0.077	0.004	1	14.38	**0.0006**
		Outside	0.053	0.004			
	Cattery	Senior	0.074	0.008	3	2.92	**0.0497**
		Overweight	0.095	0.007			
		Female	0.056	0.004			
		Male	0.052	0.004			
Stool weight (kg)	Treatment	Control	0.025	0.002	1	14.77	**0.0005**
		CEMS	0.018	0.002			
	Week	1	0.022	0.002	1	1.77	0.1924
		2	0.021	0.002			
	Litter tray location	Inside	0.030	0.002	1	12.73	0.0012
		Outside	0.010	0.002			
	Cattery	Senior	0.014	0.003	3	0.57	0.6325
		Overweight	0.030	0.004			
		Female	0.016	0.002			
		Male	0.026	0.003			

**Table 3:** Effect of treatment, week, litter tray location and cattery, on the type of elimination, scoring of stools and scoring of urine spots. For the type of elimination, score 0 = no elimination; 1 = urine only; 2 = stools only; 3 = urine plus stools. For the scoring of urine spots and stools, score 0 = no stool or urine spot; 1 = one to two stools or urine spots; 2 = two to three stools or urine spots; 3 = more than three stools or urine spots. Significant differences are highlighted in **bold**.

Variable	Effect		N	0	Score 1	2	3	DF	F	*p*-Value
Type of elimination	Treatment	Control	148	18	41	17	72	1	5.02	**0.0251**
		CEMS	148	22	57	10	59			
	Week	1	148	17	49	13	69	1	1.30	0.2545
		2	148	23	49	14	62			
	Litter tray location	Inside	168	13	39	22	94	1	7.40	**0.0065**
		Outside	128	27	59	5	37			
	Cattery	Senior	56	9	24	0	23	3	1.69	0.6387
		Overweight	64	7	18	2	37			
		Female	88	10	31	10	37			
		Male	88	14	25	15	34			
Scoring of stools	Treatment	Control	148	59	24	50	15	1	8.80	**0.0030**
		CEMS	148	79	26	32	11			
	Week	1	148	66	25	46	11	1	1.04	0.3072
		2	148	72	25	36	15			
	Litter tray location	Inside	168	52	31	61	24	1	9.59	**0.0020**
		Outside	128	86	19	21	2			
	Cattery	Senior	56	33	6	16	1	3	1.92	0.5893
		Overweight	64	25	13	20	6			
		Female	88	41	19	21	7			
		Male	88	39	12	25	12			
Scoring of urine spots	Treatment	Control	148	35	35	43	35	1	0.07	0.7984
		CEMS	148	32	38	48	30			
	Week	1	148	30	41	45	32	1	0.10	0.7567
		2	148	37	32	46	33			
	Litter tray location	Inside	168	35	30	62	41	1	2.03	0.1544
		Outside	128	32	43	29	24			
	Cattery	Senior	56	9	18	13	16	3	6.98	0.0725
		Overweight	64	9	4	27	24			
		Female	88	20	24	30	14			
		Male	88	29	27	21	11			

## Data Availability

The data presented in this study are available on request from a.cozzi@group-irsea.com. The data are not publicly available due to potential patent pending.
